# High‐throughput identification and diagnostics of pathogens and pests: Overview and practical recommendations

**DOI:** 10.1111/1755-0998.12959

**Published:** 2018-12-04

**Authors:** Leho Tedersoo, Rein Drenkhan, Sten Anslan, Carmen Morales‐Rodriguez, Michelle Cleary

**Affiliations:** ^1^ Natural History Museum and Institute of Ecology and Earth Sciences University of Tartu Tartu Estonia; ^2^ Institute of Forestry and Rural Engineering Estonian University of Life Sciences Tartu Estonia; ^3^ DIBAF University of Tuscia Viterbo Italy; ^4^ Southern Swedish Forest Research Centre Swedish University of Agricultural Sciences Alnarp Sweden

**Keywords:** community ecology, crop disease, forest pathology, high‐throughput sequencing, metabarcoding, metagenomics, molecular diagnostics, plant pathogens, sentinel plantations

## Abstract

High‐throughput identification technologies provide efficient tools for understanding the ecology and functioning of microorganisms. Yet, these methods have been only rarely used for monitoring and testing ecological hypotheses in plant pathogens and pests in spite of their immense importance in agriculture, forestry and plant community dynamics. The main objectives of this manuscript are the following: (a) to provide a comprehensive overview about the state‐of‐the‐art high‐throughput quantification and molecular identification methods used to address population dynamics, community ecology and host associations of microorganisms, with a specific focus on antagonists such as pathogens, viruses and pests; (b) to compile available information and provide recommendations about specific protocols and workable primers for bacteria, fungi, oomycetes and insect pests; and (c) to provide examples of novel methods used in other microbiological disciplines that are of great potential use for testing specific biological hypotheses related to pathology. Finally, we evaluate the overall perspectives of the state‐of‐the‐art and still evolving methods for diagnostics and population‐ and community‐level ecological research of pathogens and pests.

## 1 INTRODUCTION

Globalization and international trade of plants have greatly accelerated the frequency and magnitude of pest and pathogen invasions to agroforestry systems leading to novel encounters with plant hosts (Lenzen et al., 2012; Liebhold, Brockerhoff, Nuñez, Wardle, & Wingfield, 2017). In some rare cases, these invasive antagonists have caused large‐scale transformations of native ecosystems and changed the ecological dynamics through local and regional extinction of native host species (Prospero & Cleary, 2017) and collapses of ancient civilizations (Santini, Liebhold, Migliorini, & Woodward, 2018). In addition, climate change facilitates the probability of establishment of introduced pests and pathogens and promotes range expansion of existing populations (Seidl et al., 2017). Botanical gardens and early warning sentinel systems represent means to identify new and emerging risks to natural plant communities and to improve surveillance globally (Barham, 2016; Vettraino et al., 2015).

Besides economic damage and disease in plants and animals including humans (Seyedmousavi et al., 2018), pathogens and pests play a key role in maintaining diversity and primary productivity in natural ecosystems (Bagchi et al., 2014; Maron, Marler, Klironomos, & Cleveland, 2011). This Janzen–Connell phenomenon occurs mainly through herbivory or root decay by hexapods or microbial pathogens that are specialized on the dominant plant species and selectively increase their mortality at the seedling stage (Liang et al., 2016).

Traditionally, microbial organisms including pathogens have been identified based on symptoms of disease or culture morphology, whereas detection of pests usually relies on morphological characters of representative individuals. Many obligate intracellular pathogens do not grow in pure culture and never form reproductive structures, which render their detection and identification difficult. Furthermore, both microbial pathogens and animal pests may exhibit high intraspecific variability or comprise cryptic species that may strongly differ in niche and aggressiveness (Ashfaq & Hebert, 2016; Tuda, Kagoshima, Toquenaga, & Arnqvist, 2014). Within biological species, genotypes or races may also differ in their pathogenicity (Barnes et al., 2016; Brasier & Kirk, 2010), sometimes depending on the presence of accessory pathogenicity loci and chromosomes (Möller & Stukenbrock, 2017). These inter‐ and intraspecific differences emphasize the importance of precise detection of the organisms at the level of species and pathotypes or strains therein.

Rapid and accurate identification of pathogenic microorganisms and pests is essential for detection and employment of appropriate mitigation measures (Comtet, Sandionigi, Viard, & Casiraghi, 2015). Since the early 1990s, molecular methods brought a revolution into our understanding about the identity and autecology of microbial species and shed light into the population structure and community composition of microbiome, including pathogens (Abdelfattah, Malacrinò, Wisniewski, Cacciola, & Schena, 2018; Grünwald, McDonald, & Milgroom, 2016; Hyde et al., 2013; Pace, 1997). Several reviews provide an overview about use of the early molecular techniques for the identification of pests, biocontrol agents and microbial pathogens (Gher‐bawy & Voigt, 2010; Kashyap, Rai, Kumar, Chakdar, & Srivastava, 2017b; Levesque, 2001; McCartney, Foster, Fraaije, & Ward, 2003; Sankaran, Mishra, Ehsani, & Davis, 2010; Schaad, Jones, & Chun, 2001). Information about more recent high‐throughput methods and analysis protocols is highly scattered, with emphasis on the overall concept (Bik et al., 2012), genetic markers (Mendes, Garbeva, & Raaijmakers, 2013), specific methods (Oulas et al., 2015) or particular organism groups such as plants and animals (Deiner et al., 2017; Taberlet, Coissac, Pompanon, Brochmann, & Willerslev, 2012), insects (Wachi, Matsubayashi, & Maeto, 2018), fungi (Dickie & St. John, 2016; Nilsson et al., 2018; Tedersoo & Nilsson, 2016), bacteria (Lagos et al., 2015; Pollock, Glendinning, Wisedchanwet, & Watson, 2018) and viruses (Adams & Fox, 2016; Mokili, Rohwer, & Dutilh, 2012). Apart from viruses, only Mendes et al. (2013), Geisen (2016) and Abdelfattah et al. (2018) focused on high‐throughput identification of prokaryotes, protists and fungi including examples from pathogens.

**Figure 1:**
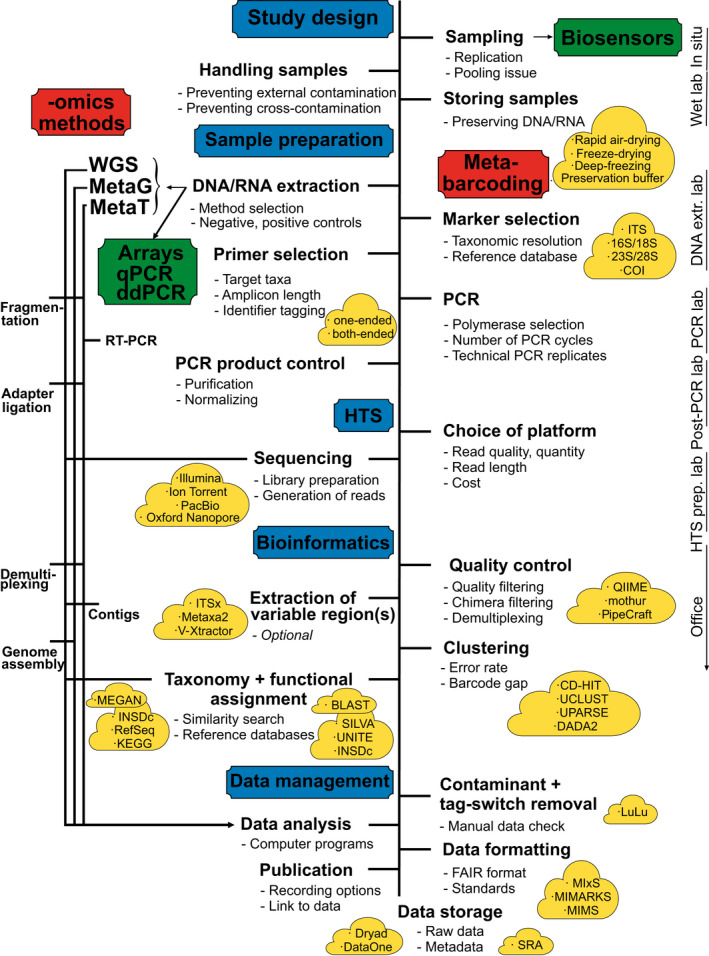
Schematic overview of a HTS‐based study from sampling through molecular analysis, bioinformatics, publishing and databasing. The most relevant and widely used platforms and methods are indicated [Colour figure can be viewed at wileyonlinelibrary.com]

This article aims to provide an overview about cutting‐edge molecular methods available for identification of plant pathogens and pests, focusing on high‐throughput identification at the species level, but also visiting genotype‐ and population‐level methods whenever appropriate for diagnosis. Based on research pitfalls and our experience, we offer practical recommendations for the analysis work flow from sampling design through molecular analysis, bioinformatics analysis, taxonomic and functional assignment and statistics (Figure 1). We illustrate the analysis steps and major achievements with examples from pathogens and pests. Finally, we provide a synthesis about the perspectives of population‐ and species‐level analyses for monitoring and efficient diagnostics of pests and pathogens.

## 2 THE EMERGING METHODS

In the last 15 years, researchers have taken advantage of the rapid development of high‐throughput molecular identification methods to characterize the enormous diversity of microbial life aboveground and belowground. These methods enable identification of thousands of taxa per sample from hundreds of samples simultaneously and facilitate concurrent focus on any groups of organisms and viruses (Bork et al., 2015; Knief, 2014; Mendes et al., 2013; Uroz, Buee, Deveau, Mieszkin, & Martin, 2016). Based on their technical aspects, high‐throughput identification methods can be divided into PCR‐based quantification methods, hybridization‐based methods (e.g., microarrays), second‐generation fingerprinting methods (e.g., RAD‐seq) and sequence‐based methods, for example, metabarcoding, (meta)genomics and (meta)transcriptomics. The first and most influential examples of these methods and their applications in pathogens and pests are concluded in Table 1.

**Table 1:** Examples of high‐throughput identification studies that focus on or include pathogens or pests

NGS technology	Sampling area, country	Sampled host species and substrate	Targeted taxa	Target DNA marker	Primers	Main results	Reference
*Studies targeting pathogens*							
Sanger: MetT	Global	*Homo sapiens: *faeces	RNA viruses	All	None	Plant viruses prevail; humans may act as vectors	Zhang et al. (2005)
454: MetB	Pakistan	Orthoptera, Gastropoda: faeces	Plants	trnL (mini)	g + h	Different plant species prevail in diet of animal pests	Valentini et al. (2009)
454: MetT	Poland + Lithuania	*Lycoperiscon esculentum: *leaves	RNA viruses	All	None	Several previously unrecognized viruses detected	Adams et al. (2009)
Illumina: MetT	Peru	*Manihot esculenta: *leaves	SPFMV, SPCSV viruses	siRNA	None	Sequence of whole genomes using siRNA method	Kreuze et al. (2009)
454: MetT	CA, USA	*Vitis vinifera: *leaves	RNA viruses	All	Various	Sequence of whole genomes from known and unknown viruses	Al Rwahnih, Daubert, Golino, and Rowhani (2009)
454: WGS	Unknown	*Phytophthora* 4 spp.: cultures	*Phytophthora*	All	None	Host jumps are followed by rapid genome evolution in repeat‐rich regions	Raffaele, Farrer, Cano, Studholme, and MacLean (2010)
454: MetT	OK, USA + Costa Rica	15 angiosperm families: leaves	dsRNA viruses	All	RT–PCR primer	11 known virus families; thousands of novel host‐specific viruses	Roossinck et al. (2010)
Illumina: MetB	WA, Australia	17 wild plant species: leaves	ssRNA Viruses	All	Oligo‐d (T) primers for RT–PCR	Multiple novel viruses describe	Wylie, Luo, Li, and Jones (2012)
454: MetB	Italy	*Phytophthora:* mock community	Oomycetes: *Phytophthora* spp.	ITS1	ITS6 + ITS7	HTS method suited for detection of *Phytophthora* spp.	Vettraino, Bonants, Tomassini, Bruni, and Vannini (2012)
Ion Torrent: MetG	South Africa	*Eucalyptus grandis*: leaf, petiole, twig, wood	Fungi	ITS1	ITS1F^a^ + ITS2^a^	Community dominated by *Dothidiomycetes* that harbour well‐known plant pathogens.	Kemler et al. (2013)
454: MetB	Finland	*Picea abies: *stumps	Basidiomycota	ITS	ITS1F^a^ + ITS4B^a^	*Phlebiopsis gigantea* biocontrol does not affect fungal composition, lost 1 year after inoculation; *Heterobasidion* undetected	Terhonen et al. (2013)
Illumina: MetG	China	*Hexapoda: *mixed specimens	Hexapoda	None (mtDNA enriched)	None	High‐resolution identification of arthropods	Zhou et al. (2013)
Illumina: MetB	Panama	Woody plants: leaves	Bacteria	16S rRNA	799F + 1115R; PCRII_for + PCRII_rev	Communities dominated by a few core microbiome taxa	Kembel et al.. (2014)
454: MetB	Martinique, France	Arthropod predators	Metazoa	COI (mini)	Uni‐MinibarF1 + R1	Multiple insect pests make a strong contribution to diet	Mollot et al. (2014)
454: MetB	Spain	Soil, water	*Phytophthora*	ITS1	18Ph2F + 5.8S−1R	Greater species and phylogenetic richness in water samples	Catala, Pérez‐Sierra, and Abad‐Campos (2015)
Illumina: RAD‐seq	Germany	*Fusarium graminearum: *cultures	*Fusarium graminearum*	None	None	High levels of divergence in all populations	Talas and McDonald (2015)
Illumina: MetB	Germany	*Arabidopsis thaliana *tissues	Bacteria, Fungi, Oomycetes	16S rRNA ITS1. ITS2	2 pairs for each group	Pathogens regulate microbiome diversity; fungal and oomycete antagonism to bacteria	Agler et al. (2016)
Illumina: MetB	WA, USA	*Populus trichocarpa: *leaves	Fungi	ITS1	ITS1F^a^ + ITS2^a^	Detection of species suppressing and facilitating rust infection	Busby, Peay, and Newcombe (2016)
454: MetB	Primorye, Russia	*Fraxinus * *mandshurica: *leaf, leaflets, rachises	Fungi (incl. *Hymenoscyphus* spp.)	ITS2	gITS7 + ITS4	Fungal composition similar in infected and uninfected leaves; *H. fraxineus *detected from 33% of samples	Cleary et al. (2016)
PacBio: MetaB	Mexico	*Coffea arabica * *+Hemileia* * vastatrix*: pustules	Fungi	ITS	ITS1F^a^ +ITS4	Communities diverse, differ geographically; potential biocontrol agents detected	James, Marino, Perfecto, and Vandermeer (2016)
Illumina: MetT	North America	Cultures of five fungal pathogens	RNA viruses	All	None	Tens of novel mycoviruses that may have biocontrol properties	Marzano et al. (2016)
Illumina: WGS	Variable	Erysiphales spp.: cultures	Erysiphales (Fungi)	None	None	Interspecific hybridization causes raise of novel pathogens	Menardo et al. (2016)
ON: WGS	Guinea	*Homo sapiens*: tissue samples	Ebola virus	None	None	Virus genome sequenced and detected in <60 min.	Quick et al. (2016)
Illumina: MetB	France	*Raphanus sativus*	Bacteria, Fungi	16S, gyrB, ITS1	515F+806b aF64+aR353 ITS1F^a^+ITS2^a^	*Alternaria* infection alters plant microbiome	Rezki et al. (2016)
Illumina: MetB	Estonia	Forest nursery soils	Oomycetes	ITS	ITS1Oo + ITS4ngs	HTS conditions suitable for identification of oomycetes	Riit et al. (2016)
454: MetB	Great Britain	*Quercus *spp.: bark, wood	Bacteria	16S rRNA	341f + 805r	Composition differs in healthy and infected tissue by acute oak decline	Sapp et al. (2016)
454: MetB	SA, Canada	4 crops: roots and soil	Fungi	ITS1	ITS1F^a^ + ITS2^a^	Pathogens become dominant after crop rotations with legumes	Bainard et al. (2017)
454: MetB	Spain	*Quercus ilex*: roots, soil	Oomycetes, esp. *Phytophthora*	ITS1	ITS6^a^ + 5.8S−1R	*Phytophthora *sp. nov dominates	Catala, Berbegal, Pérez‐Sierra, and Abad‐Camposa (2017)
454: MetB	Norway	*Fraxinus excelsior: *leaves, petioles	Fungi (incl. *Hymenoscyphus* spp.)	ITS1, ITS2	ITS5^a^ + ITS2^a^; gITS7 + ITS4	Fungal communities differ by season and infection rate; few reads of *Hymenoscyphus* spp. in ITS1 data	Cross et al. (2017)
454: MetB	Italy	Bark beetles: *Orthotomicus erosus, Xyleborinus saxesenii*	Fungi	ITS2	ITS3^a^ + ITS4	Bark beetles carry pathogenic fungi with exported timber	Malacrino et al. (2017)
454: MetB	Finland	*Betula pendula*: leaves	Fungi	ITS2	fITS7^a^ + ITS4	No effect of tree diversity and neighbourhood on pathogens	Nguyen et al. (2017)
Ion torrent: ddRAD sequences	Multiple	*Hymenoscyphus fraxineus*: ascocarps, isolates; *H. albidus*: isolates	Fungi: *H. albidus, H. fraxineus*	None	None	High genetic variation in E Asia compared to Europe; E Russia a likely source area for Europe; possibility to assign strain to a population	Sønstebø et al. (2017)
Illumina: MetB	France	*Arabidopsis thaliana: *tissues	Bacteria	gyrB	aF64+aR353	Pathogens co‐occur in diseased plants, differ in roots and leaves and seasonally	Bartoli et al. (2018)
PhyloChip	Netherlands	*Arabidopsis thaliana: *roots	Bacteria, Fungi	None	None	Pathogen‐induced root microbes induce systemic resistance in offspring	Berendsen et al. (2018)
Illumina: MetB	Slovenia, Germany	*Centaurea *spp.: rhizosphere	Nematodes	18S	3NDf + 1132rmod	MetB outperforms qPCR in nematode identification	Geisen et al. (2018)
ON: MetG	New Zealand	*Rattus norvegicus*: gut	All	None	None	Diet consists of various plants and insects incl. pests	Pearman et al. (2018)
*Studies including pathogens*							
454: MetT	Europe	Agricultural soils	All (esp. ammonia oxidizers)	None (AmoA)	None (various for AmoA)	Archaea are predominate ammonia oxidizers	Leininger et al. (2006)
454: MetB	Various	Various soils	Bacteria, Archaea	18S	787f + 1492rm	Richness and composition differ in agricultural and forest soils; pathogens undistinguished	Roesch et al. (2007)
454: MetG	China	Vector insect	*Candidatus* *Liberibacter asiaticus*	None	None	Plant pathogen genome recovered from mixed DNA samples	Duan et al. (2009)
454: MetB	France	6 tree plantations: soil	Fungi	ITS1	ITS1F^a^ + ITS2^a^	Putative pathogen Ceratobasidiaceae sp. dominant in all sites	Buee et al. (2009)
454: MetB	KS, USA	*Quercus macrocarpa*: leaves in rural versus urban sites	Fungi	ITS1	ITS1F^a^ + ITS2^a^	Both pathogens and endophytes common, habitat effect	Jumpponen and Jones (2009)
454: MetB	Americas	Soil	Bacteria	16S	27F + 338R	pH drives soil bacterial composition on a continental scale; pathogens undistinguished	Lauber, Zhou, Gordon, Knight, and Fierer (2010)
454: MetB	FL, USA + unknown	Individuals extracted from soil	Nematoda	18S; 28S	NF1 + 18Sr2b^a^; D3a + D3b	Two markers recover nearly all taxa	Porazinska et al. (2009)
454: MetB	Costa Rica	Forest soil	Eukaryotes	18S; 28S	SSUF04 + SSUR22; NF1 + 18Sr2b^a^	HTS of 18S and 28S rRNA genes can be used for eukaryote diversity studies	Creer et al. (2010)
454: MetB	Global	Indoor dust	Fungi	28S	LROR‐F + LR5F	Indoor dust reveals predominance of microfungi incl. plant pathogens	Amend, Seifert, Samson, and Bruns (2010)
454: MetB	Americas	Soil	Eukaryotes (esp. protists)	18S	F515^a^ + R1119^a^	Distribution of protist phyla depends on climate; pathogens undistinguished	Bates et al. (2013)
454: MetB	KS, USA	Individuals extracted from soil	Nematoda	18S	NF1 + 18Sr2b^a^	Quantification of taxa semiquantitative at best	Darby, Todd, and Herman (2013)
Illumina: MetB	NY, USA	Green roof and city park soil	Fungi	ITS1	ITS1F^a^ + ITS2^a^	Fungal composition differs, pathogens undistinguished	McGuire et al. (2013)
Illumina: MetB	Germany	Grassland soil	Fungi	ITS1	ITSFI2^a^ + ITS2^a^	Illumina HTS can be used to recover fungal diversity	Schmidt et al. (2013)
454: MetB	NC, TN, USA	3 tree species: roots	Bacteria, Fungi	16S, 18S, ITS, 28S	various	Soil origin explains endophytes better than host species; pathogens commonly detected	Bonito et al. (2014)
454: MetB	Portugal	*Vitis vinifera*: leaves	Bacteria, Fungi	16S, ITS2, 28S	V6F, V6R; ITS3^a^ + ITS4; D2F^a^ + D2R	Fungal richness declines but bacterial richness increases with time	Pinto et al. (2014)
454: MetB	Global	Soil	Fungi	ITS2	ITS3NGSmix + ITS4ngs	Plant pathogen richness peaks in tropics	Tedersoo et al. (2014)
454: MetB	Costa Rica	Reared individuals	Lepidoptera	COI	LepF1 + LepR1	HTS can be used in single‐specimen barcoding	Shokralla et al. (2014)
454: MetB	Italy	*Olea europaea*: fruits, leaves	Fungi	ITS2	ITS3^a^ + ITS4	Low diversity and high *Colletotrichum *abundance in rotten fruits	Abdelfattah, Nicosia, Cacciola, Droby, and Schena (2015)
Illumina: mtMetG	Spain	Coleoptera*: *bodies	Coleoptera	COI, etc.	None	Larval species and phylogenetic diversity greater in subsoil	Andujar et al. (2015)
Illumina: MetB	AK, USA + Canada	*Populus balsamifera*: leaves	Fungi	ITS1	ITS1FI2^a^ + ITS4	Proportion of pathogens declines northwards	Balint et al. (2015)
454: MetT	Europe	Soil	Eukaryotes	None	None, 18S extracted	High protist diversity incl. many pathogens	Geisen et al. (2015)
Illumina: MetB	USA	Dust	Fungi	ITS1	ITS1F^a^ + ITS2^a^	Spore dispersal prediction for pathogens and other fungi	Grantham et al. (2015)
454: MetB	FL, USA	Scolytidae bark beetles: mycangia	Fungi	ITS2	gITS7 + ITS4	Composition differs among species; several yeasts and plant pathogens besides mutualists	Kostovcik et al. (2015)
454: MetB	Italy	Fruit fly: *Bactrocera oleae*	Fungi	ITS2	ITS3^a^ + ITS4	Fruit fly carries spores of both pathogens and biocontrol agents; males and females differ	Malacrino, Schena, Campolo, Laudani, and Palmeri (2015)
Illumina: MetB	Costa Rica	1,010 trapped individuals	Arthropoda	COI	Ill_LCO1490 + Ill_C_R; Ill_B_F + Ill_HCO2198	Low match between morphology, Sanger sequencing and HTS	Shokralla et al. (2015)
454: MetB	Italy	*Fragaria vesca: *fruits, leaves	Fungi	ITS2	ITS3^a^ + ITS4	Fungicides reduce the dominant species *Botrytis cinerea*	Abdelfattah, Wisniewski, Nicosia, and M.G., Cacciola, S.O., & Schena, L. (2016)
454: MetB	Estonia	Forest soil	Eukaryotes	ITS2	ITS3NGSmix + ITS4ngs	Pathogen composition is relatively most affected by plant neighbourhood	Bahram et al. (2016)
Illumina: MetB	Panama	Plantation forest soil	Fungi	28S	NL1 + NL4	Pathogens respond more strongly to vegetation than saprobes and mutualists	Kivlin and Hawkes (2016)
Illumina: MetG	Brazil	Predator arthropods: gut	All	None	None	Insect pests are important food sources	Paula et al. (2016)
454: MetB	Estonia + Finland	Forest soil	Eukaryotes	ITS2	ITS3NGSmix + ITS4ngs	Plant pathogens respond negatively to soil C:*N* ratio; nematodes have site‐specific drivers	Tedersoo, Bahram, et al. (2016)
454: MetB	The Netherlands	Agricultural soils	Fungi	ITS2: ^13^C enriched	ITS9^a^ + ITS4	Pathogen: mycorrhiza ratio of active community declines with succession	Hannula et al. (2017)
Illumina: MetG	Great Britain	*Solanum tuberosum: *rot	All	None	None	Detection of taxonomic and functional profile of potato rot spots	Doonan et al. (2017)
Illumina: MetB	MN, USA	Agricultural soil	Fungi	ITS2	ITS1F^a^ + ITS2^a^	Pathogens are relatively more abundant in high‐biomass plant communities	Cline et al. (2017)
Ion Torrent: MetB	Greenland	Tundra soil	Fungi	ITS2	fITS7^a^ + ITS4	Pathogens were more prevalent in highly stressed habitats	Grau et al. (2017)
Illumina: MetB	North America	*Helianthus annuus: *roots, leaves, seeds	Bacteria, Fungi	16S; ITS1	515F + 806r; ITS1F^a^ + ITS2^a^	Proportion of pathogens greater in older than recent varieties	Leff, Lynch, Kane, and Fierer (2017)
Illumina: MetB	NY + MA, USA	Forest soil	Fungi	ITS2	fITS7^a^ + ITS4	Pathogen richness is greater in *Alliaria petiolata* infested sites	Anthony, Frey, and Stinson (2017)
454: MetB	Estonia	*Pinus sylvestris* forest soil	Eukaryotes	ITS2	ITS3NGSmix + ITS4ngs	Plant pathogen richness increase at higher soil moisture	Hiiesalu, Bahram, and Tedersoo (2017)
PacBio: MetB	Papua New Guinea + Estonia	Forest and nursery soil	Eukaryotes (esp. fungi, oomycetes)	18S, ITS, 28S	Multiple	Longer amplicons: higher resolution, less artefactual taxa; full ITS‐based identification of oomycetes	Tedersoo et al. (2017)
Illumina: MetB	S Europe	Bat *Miniopterus schreibersii: *faeces	Animals	COI, mt16S	ArtF1c + ArtR2c; Coleop16Sc + Coleop16Sd	Pests constitute >50% of prey	Aizpurua et al. (2018)
Illumina: MetB	SA, Australia	Soil in successional habitats	Fungi	ITS (ITS1 analysed)	ITS1F^a^ + ITS4	Pathogen abundance declines with ecosystem naturality	Yan et al. (2018)

MetB, metabarcoding; MetG, metagenomics; metT, metatranscriptomics; ON, Oxford Nanopore; WGS, whole‐genome sequencing.

a Poorly performing primer(s).

### 2.1 Quantification methods

Taxon‐specific primers and quantitative PCR (qPCR) methods have been used for two decades to determine the relative and/or absolute abundance of pathogenic organisms (Sanzani, Nicosia, & M.G., Faedda, R., Cacciola, S.O., & Schena, L., 2014; Schena, Nigro, Ippolito, & Gallitelli, 2004). Recent technical advances enable running thousands of sample and template combinations in parallel. For example, Muurinen et al. (2017) performed simultaneous replicated screening for hundreds of antibiotics resistance genes.

Droplet digital PCR (ddPCR) is based on microfluidics technology, which separates the amplification reaction into >20,000 individual droplets and allows absolute quantification of DNA from up to four target organisms or genes simultaneously, with a detection limit of 10^−5^ relative abundance (Hindson et al., 2011). Dreo et al. (2014) showed much greater precision of ddPCR for quantification of two bacterial plant pathogens with optimal and suboptimal primers compared with ordinary qPCR. Currently, ddPCR can be run in 96‐well and 384‐well plates, but it is technically possible to increase the throughput of samples. It may be also possible to increase the number of fluorescent dyes to be able to multiplex >4 reactions. Overview of the methodology and use in pathology is reviewed in Gutierrez‐Aguirre, Rački, Dreo, and Ravnikar (2015).

Quantification of marker or functional genes is possible by spiking approach combined with HTS identification methods. For spiking, known quantity of control DNA or individuals is added to the sample prior to DNA/RNA extraction and the quantity of target organisms or genes is detected based on the relative amount of obtained sequences (Pochon, Bott, Smith, & Wood, 2013; Tkacz, Hortala, & Poole, 2018). In theory, spiking allows absolute quantification of the DNA marker content of any pathogenic organism or gene, but this method has been little tested thus far. Differences in sequence length, G + C/A + T content, DNA secondary structure, etc. (see Technical biases below), may all affect accuracy of the spiking approach.

### 2.2 Microarrays

Microarray technology is based on accommodation of multiple target‐template hybridization reactions onto small chips using robotics technologies to generate arrays and perform multiple hybridization reactions simultaneously. Microarrays have been widely used for species diagnosis, detection of functional genes and gene expression (Sessitsch et al., 2006). Diagnostic microarrays were the earliest high‐throughput identification methods that enabled targeting specific pre‐selected taxa of viruses, bacterial and fungal pathogens and pests at species level (Lee et al., 2013; Szemes et al., 2005; Wilson et al., 2002). The first diagnostic (macro)arrays for selected plant pathogenic fungi and oomycetes included just >10 species and enabled quantification of pathogens present at low abundance (<0.1% in mixed DNA samples; Lievens et al., 2005; Szemes et al., 2005). By combining classical antagonism tests and high‐density microarrays comprising >106 probes, Mendes et al. (2011) identified rhizosphere microbial taxa and particular genes that are antagonistic to a fungal pathogen *Rhizoctonia solani*. PhyloChip‐based analyses revealed that plant infection by pathogenic oomycete *Hyaloperonospora arabidopsidis* enhanced growth of rhizosphere microbes, which triggered systemic resistance and reduced damage in the plants’ offspring (Berendsen et al., 2018).

While early microarrays used PCR‐amplified templates, fine tuning of sensitivity enabled to detect taxa from genomic DNA (DeAngelis et al., 2011). Microarrays also enable to detect single nucleotide polymorphisms (SNPs), which allow genotyping of plant pathogens and detection of aggressive strains (Lievens, Claes, Vanachter, Cammue, & Thomma, 2006). Although reusable microarrays are cheap to run, provide highly sensitive results rapidly and suffice for monitoring the presence and abundance of specific pathogenic taxa and pathogenicity‐related genes from complex samples, their major disadvantage is missing the large proportion of species and functions present in the targeted environment and non‐optimal stringency in various probes (Sessitsch et al., 2006). Therefore, microarrays have been replaced by high‐throughput sequencing (HTS) methods in the last decade.

### 2.3 HTS methods for identification of species

High‐throughput sequencing represents several new and emerging technologies that fundamentally differ in their ways of recording nucleotides. Furthermore, these methods exhibit substantial differences in throughput, read length, accuracy and technical biases (Knief, 2014; Reuter, Spacek, & Snyder, 2015). During the first five years in the market, HTS platforms usually evolve rapidly in terms of throughput, data quality and reduction in analytical costs, but technical constraints become limiting soon thereafter. Fundamentally new HTS methods are announced almost every year, but a fraction of these gain public attention and approximately half of those appear in the market (Heather & Chain, 2016). Table 2 provides an overview of widely used HTS platforms.

**Table 2:** Cons and pros of sequencing platforms

Platform	Read length (average: max; kb)	Error rate (%, per bp): main issues	Throughput (10^6^ reads)	Cost (run: library prep.; EUR)	Optimal (suboptimal) use
454: GS‐FLX (discontinued)	0.7–1:1	0.1: homopolymer indels, end	1.2 (run)	5,000:200	MetB (MetG, WHG)
Ion Torrent: PGM	0.4:0.45	0.5–1.5: homopolymer indels, start, end	5 (chip 318)	1,000:150	MetB (MetG, WHG)
Ion Torrent: Gene Studio S5^a^	0.5–0.6:0.6	0.5–1.5: homopolymer indels, start, end	12 (chip 530)	n.d.: n.d.	MetB (MetT, MetG, WHG)
Illumina: MiSeq	2 × 0.3 (paired‐end: 0.58)	0.01–0.1: substitutions, end	20 (lane)	1,500:50–100	MetB (MetG, MetT, WHG)
Illumina: HiSeq	2 × 0.25 (paired‐end: 0.48	0.01–0.1: substitutions, end	300 (lane)	4,500:50–100	MetG, MetT, WHG (MetB)
BGISEQ‐5002	2 × 0.15 (paired‐end 0.28), single‐end: 0.4	0.08–0.5: long switch	600 (lane)	*n*.d.: *n*.d.	MetG, MetT, WHG
PacBio: Sequel	30:100	13 (raw); <0.1 (10× consensus): homopolymer indels	0.4 (SMRT cell)	1,500:300	MetB, WHG
Oxford Nanopore: MinION	10–100:800	15 (raw); 3–5 (bidirectional): various	0.1–0.35 (flow cell =run)	500:50	WHG (MetB)
Oxford Nanopore: PromethION^a^	n.d.: n.d.	15 (raw); 3–5 (bidirectional): various	100 (run)	n.d.: n.d.	WHG (MetB, MetG, MetT)

a Service not available as of October 2018; hence, the values are approximate

Service offered through collaborative contract as of October 2018; prices and terms negotiable

The first commercially available HTS method, 454 pyrosequencing (Roche Diagnostics, Basel, Switzerland), was developed in early 2000s. The 454 technology was >100‐fold cheaper (10^−2^ EUR/read) than Sanger sequencing, and the analysis chemistry was rapidly optimized to provide high‐quality reads from 50 to 700–1,000 bases at 1.2 million read throughput (Reuter et al., 2015). The 454 technology was rapidly adopted by microbial ecologists who performed ground‐breaking discoveries about the ultra‐high diversity of prokaryotes (Leininger et al., 2006; Sogin et al., 2006). Anecdotally, much of the diversity turned out to be analytical artefacts, indicating the need for careful quality control and optimization of both sample preparation and analytical steps (Huse, Huber, Morrison, Sogin, & Mark Welch, 2007). Separation of artefacts from rare taxa is still the greatest issue of all HTS technologies. Soon after these pioneering prokaryote studies, 454 pyrosequencing was implemented to identify eukaryotes and to separate potentially pathogenic taxa from other guilds based on taxonomic information from indoor environment, animal samples, soil and foliage (Buee et al., 2009; Jumpponen & Jones, 2009; Luna et al., 2007; McKenna et al., 2008; Wegley, Edwards, Rodriguez‐Brito, Liu, & Rohwer, 2007). Several years after implementation, the 454 method was used to identify macroorganisms such as plants from mammal and hexapod diet (Valentini et al., 2009) and animals including parasitic nematodes and other pests (Creer et al., 2010; Porazinska et al., 2009; Table 1).

The Illumina (www.illumina.com) and Ion Torrent (www.iontorrent.com) technologies replaced 454 in the early 2010s because of greater throughput at lower costs. Nonetheless, the Ion Torrent is continuously haunted by short read length (up to 450 bp) and fluctuating sequence quality, which has limited its use in analysis of soil and plant samples (see Kemler et al., 2013). Compared with the 454 platform, the Illumina technology provides up to 3,000‐fold greater throughput, several times greater accuracy and possibility to sequence reads of up to 550 bp (2 × 300 paired‐end option) at relatively low cost, 10^−5^–10^−4^‐EUR/read. Generation of self‐chimeric sequences and long artefactual inserts or deletions represents the main shortfall of Illumina sequencing compared to other platforms (Tedersoo, Anslan, et al., 2015). At present, Illumina sequencing is by far the best option for short DNA/RNA barcodes and metagenomics, considering sequence quality and analytical costs of library preparation and sequencing (Knief, 2014). It will undoubtedly remain the most widely used HTS method by the end of this decade in spite of only negligible technological improvements since 2015. The ultra‐high throughput of Illumina sequencing allows analysis of >1,000 samples in a single run at sufficient sequencing depth (Zinger et al., 2017). Illumina sequencing revealed that growing rotations of legume crops greatly increase the pathogen load in soils, with several‐year legacy effects (Bainard et al., 2017). Cline et al. (2017) showed that relative abundance of soil pathogens increases with plant biomass in grasslands. In 2015, a paired‐end ultra‐HTS platform BGISEQ (www.seq500.com/en/), which is similar to the Illumina platform, was released. So far, it has been used for metagenomic detection of human pathogens (Cheng et al., 2018). Given its shorter read length (2 × 150 paired‐end or 400 bases single end), it is currently suboptimal for amplicon‐based detection and identification of organisms.

As a major technical advance, much longer DNA fragments spanning tens of kilobases can be sequenced using the Pacific Biosciences (PacBio, www.pacificbiosciences.com) and Oxford Nanopore (www.nanoporetech.com) technologies, which became commercially available in 2011 and 2015, respectively. However, both of these platforms have very high initial error rates (10%–15% per base) that have improved only marginally in the last years. In the PacBio platform, circularized DNA molecules are sequenced multiple times, reducing the error rate to a minimum (0.1%) at 9‐ to 11‐fold consensus (Tedersoo, Tooming‐Klunderud, & Anslan, 2017). Given the average raw read length of 30 kb, PacBio allows sequencing of up to 5 kb DNA fragments at satisfactory quality (Heeger et al., 2018). Sequencing of long fragments of a single molecule has become attractive in DNA barcoding; for example, Hebert et al. (2017) reported on sequencing the DNA barcode in around 10,000 arthropod specimens simultaneously. In a pioneer study, PacBio was successfully applied to identify potential mycoparasites of the coffee rust, *Hemileia vastatrix*. In general, long fragments greatly improve identification via greater taxonomic resolution of unconserved regions and phylogenetic analysis based on relatively more conserved regions (Schlaeppi et al., 2016; Tedersoo et al., 2017; Wagner et al., 2016). However, both library preparation and sequencing steps of PacBio are relatively expensive (ca. 300 EUR/library, 10^−2^ EUR/read) compared with Illumina sequencing. Thus, PacBio is the method of choice for metabarcodes >550 bp given sufficient funding.

Application of the nanopore technology in pathology and ecology in general suffers greatly from low sequence quality, although sequencing of both strands (1D^2^ flow cell) and Intramolecular‐ligated Nanopore Consensus Sequencing (INC‐Seq) technique (Li et al., 2016) have been developed. These advances reduce the error rate to 2%–5%, which is still insufficient for distinguishing among closely related species. The first applications in ecology also stress the need for longer identifier tags and avoiding clustering methods (Benitez‐Paez & Sanz, 2017; Kerkhof, Dillon, Häggblom, & McGuinness, 2017; Krehenwinkel et al., 2018). The Oxford Nanopore MinION platform is therefore mostly used as a cheap option to close gaps, resolve long repeats and merge scaffolds in genome sequencing, or perform whole‐genome resequencing. For example, Rhodes et al. (2018) sequenced multiple strains of an opportunistic human pathogen *Candida auris* and suggested (unsubstantiated though) Indian origin for the pathogenic strains. Nanopore consensus sequences have been used to acquire long DNA barcodes from multiple arthropod specimens simultaneously (Krehenwinkel et al., 2018). However, routine metabarcode‐based identification of fungi and oomycetes with the MinION device suffers from very low proportion of meaningful sequences, extremely common tag‐switching events and highly unequal sequencing depth across samples (K. Loit, K. Adamson, R. Drenkhan, M. Bahram, R. Puusepp and L. Tedersoo, unpublished manuscript). In spite of high error rate, the nanopore technology holds a great promise in disease diagnostics due to the low cost of equipment and analysis time of 1–2 days (Quick et al., 2016). The unique direct RNA sequencing option (without cDNA reverse transcription step) is of particular interest, but it requires testing for environmental samples and analytical biases.

### 2.4 Metagenomics and metatranscriptomics

PacBio and Oxford Nanopore are sometimes termed as third‐generation sequencing technologies because of long reads and possibility of excluding the PCR Step. Also, the Sanger method (Green Tringe et al., 2005) and multiple HTS methods (Frey et al., 2014) have been used for generating amplification‐free shotgun metagenomic and metatranscriptomic sequence data sets from DNA and RNA (through reverse transcription) molecules, respectively, to address taxonomic diversity of animal pathogens. Except for viruses (see below), shotgun metagenomic and metatranscriptomic studies specifically targeting plant pathogens are rare, although several projects unintentionally cover pathogens in addition to free‐living microbes and their functions (Fierer et al., 2013; Geisen et al., 2015; Hudson et al., 2015; Tedersoo, Anslan, et al., 2015; Tedersoo, Bahram, et al., 2015). More specifically, Doonan, Denman, McDonald, and Golyshin (2017) sequenced the metagenomes of soft rot spots of *Solanum tuberosum* to identify the relative abundance of multiple microbial organisms and their functional potential. The metatranscriptomic approach revealed that root colonization by the pathogenic *Rhizoctonia solani* altered rhizobacterial communities and induced expression of stress‐related genes (Chapelle, Mendes, Bakker, & Raaijmakers, 2016). A nanopore technology‐based metagenomic study revealed that the invasive *Rattus norvegicus* consume mostly plants and insects in New Zealand (Pearman et al., 2018).

No universal primers exist for viruses, rendering metagenomics and metatranscriptomics the only suitable methods for detecting previously unrecognized viruses (Mokili et al., 2012; Zhang, Breitbart, Lee, Run, & Wei, 2005). Metagenomic‐ and metatranscriptomic‐based identification of viruses has been recently reviewed in Massart, Olmos, Jijakli, and Candresse (2014) and Roossinck, Martin, and Roumagnac (2015) and Adams and Fox (2016). Roossinck et al. (2015) in particular provide information about alternative analysis work flows for single‐ and double‐stranded DNA and RNA viruses. For dsRNA viruses, metatranscriptomics of the 21–24 base fragments of silencing RNA (siRNA) has become a popular identification tool of various viruses due to ease of analysis and high detection capacity of small RNA analysis (Kreuze et al., 2009; Roossinck et al., 2015).

Because of various biases introduced by primer choice and the PCR amplification process, PCR‐free technologies offer great promise to molecular identification of organisms, particularly viruses and bacteria. In spite of generating huge amounts of sequence data, shotgun sequencing of full metagenomes or metatranscriptomes is an inefficient approach to taxonomic identification of eukaryotes (Alberdi, Aizpurua, Gilbert, & Bohmann, 2018; Tedersoo, Anslan, et al., 2015; but see Geisen et al., 2015), because only a tiny fraction of the sequences is likely to originate from relevant marker genes. Furthermore, metagenome and metatranscriptome analyses suffer from several technical problems. Because organisms differ substantially in their AT:CG ratio, genomic fragments with extreme ratios may be disfavoured in the sequence analyses, depending on analysis platform (Shakya et al., 2013). The metagenomic fragments cover random stretches of the marker genes among other genomic regions, rendering it impossible to address species‐level taxonomic richness in natural communities (Tedersoo, Anslan, et al., 2015). The marker‐based reference databases such as UNITE (Abarenkov et al., 2010) and SILVA (Quast et al., 2013) contain abundant data for relatively short rRNA gene markers, but much less taxa have full‐length reference sequences for comparison. The genomic reference databases are underpopulated and may result in misidentifications up to the level of kingdom (Korsakovsky Pond et al., 2009; Pearman et al., 2018; Tedersoo, Anslan, et al., 2015). Nonetheless, metagenomics and metatranscriptomics offer an option for identifying the taxa and characterizing their functional potential simultaneously (Bork et al., 2015; Fierer et al., 2013).

Mitochondrial metagenomics has become a high‐quality alternative in biodiversity studies of soil arthropods due to the high abundance and rapid evolution of mitochondria and the lack of PCR bias. Genomic DNA samples can be effectively enriched for mitochondrial products that are typically fragmented and sequenced on Illumina platform (Zhou et al., 2013). Gomez‐Rodríguez, Timmermans, Crampton‐Platt, and Vogler (2017) found that mitochondrial metagenomics is more sensitive to taxon recovery compared with COI metabarcoding and it allows addressing intraspecific variation and construction of more robust phylogenies. This method revealed greater species richness and phylogenetic diversity of beetles in subsoil compared with topsoil (Andujar et al., 2015). In principle, mitochondrial metagenomics could be applied to any group of eukaryotic organisms that possess these organelles. The main drawbacks include a large variation in the number of mitochondria across organisms and their different life stages as well as poor representation in reference databases (except animals).

## 3 HTS METHODS FOR IDENTIFICATION OF INDIVIDUALS

HTS methods can be used to distinguish between individuals when targeting SNPs in rapidly evolving loci, partial genomes or whole genomes (Fuentes‐Pardo & Ruzzante, 2017). These comparative genomics methods shed light into the origin, migration pathways, speciation, host shifts, co‐evolution, hybridization and horizontal gene transfer of antagonists and enable to detect virulent genotypes along with their underlying genetic mechanisms (Grünwald et al., 2016; O'Hanlon et al., 2018). Population genomics methods can also be used to detect genomic introgression from other species and evolution of new species by polyploidization and hybridization, which is a common mechanism in the rise of novel pathogenic fungi and oomycetes (Restrepo, Tabima, Mideros, Grünwald, & Matute, 2014). Common metabarcoding techniques may be unable to distinguish recent hybrids from parent taxa, because the hybrids usually carry the nuclear marker gene (haploid organisms) or mitochondrial marker gene (nearly all eukaryotes) from one of the parents or different nuclear alleles from both parents in case of diploid (incl. dikaryotic) and polyploid organisms.

The early HTS population genomics methods focused on the distribution of SNPs in certain variable regions. For example, Isola et al. (2005) used targeted 454 pyrosequencing to detect mutations underlying resistance to a drug in the human pathogen *Mycobacterium tuberculosis*. Subsequent population‐level studies targeted multiple genomic fragments flanking restriction sites across the entire fragmented genome, which is termed as restriction site‐associated DNA sequencing (RAD‐seq; reviewed in Davey et al., 2011). Several examples of using RAD‐seq in plant pathogens are given in Grünwald et al. (2016). This method revealed high genetic variability and recombination in a crop pathogen *Fusarium graminearum,* suggesting that these features facilitate rapid adaptation to resistant cultivars and biocides (Talas & McDonald, 2015). RAD‐seq also revealed several coexisting groups of a mutualistic fungus *Rhizophagus irregulare*, most of which were globally distributed (Savary et al., 2018).

With plummeting of HTS costs, partial and whole genomes of pathogenic organisms can be readily determined from pure cultures, host tissues and soil environment. The main advantage of whole‐genome sequencing (WGS) is the generation of several orders of magnitude more information about polymorphic sites and a better understanding of their linkage and occurrence in exons and introns (Grünwald et al., 2016). Low‐coverage genomes and organisms’ marker genes and functional genes can be determined from minute DNA concentrations from old herbarium specimens and roots (Tedersoo, Bahram, et al., 2016; Tedersoo, Liiv, et al., 2016; Yoshida, Burbano, Krause, Thines, & Weigel, 2014). Thus, sequencing of genomes from multiple isolates of the same species has become a common practice in microbiology (Liti et al., 2010) and more recently in plant pathology (Menardo et al., 2016; Table 1). Due to much greater genome size and organization of genetic material into multiple chromosomes, eukaryote genomes are more difficult and costly to sequence and assemble compared with these of prokaryotes. Using a shotgun metagenomic approach, Duan et al. (2009) sequenced the genome of an uncultured plant pathogenic bacterium *Candidatus Liberibacter asiaticus* from its psyllid vector. In a population genomics study, Cooke et al. (2012) detected recent evolution of a highly virulent genotype group within *Phytophthora infestans* and its genetic mechanisms of overcoming hosts’ resistance. WGS of the malaria parasite *Plasmodium viviparum* revealed substantial population divergence and endemicity on a global scale (Manske et al., 2012). WGS analyses using the pocket sequencer Oxford Nanopore MinION are becoming a routine for “real‐time” identification of strains and cryptic species in pathogenic complexes, which has been demonstrated for *Escherichia coli* and the Ebola virus (Loman, Quick, & Simpson, 2015; Quick et al., 2016).

Optimization of sample preparation and HTS protocols has enabled WGS of single cells (Lasken & McLean, 2014). McLean et al. (2013) sequenced the genome of an opportunistic human pathogen *Porphyromonas gingivalis *from several individual cells in parallel. Due to the small genome size, single‐cell sequencing may perform well for bacteria and archaea, but it is more problematic in eukaryotes. Single‐cell WGS revealed that human neurons differ in partial genome copy number and some cells have multiple mutations in specific regions (McConnell et al., 2013). Using microfluidics‐based single‐cell preparation and sequencing, Gawad, Koh, and Quake (2014) determined SNPs from leukaemia cells and shed light into the mechanisms of cancer development. Nair et al. (2014) demonstrated the potential of single‐cell sequencing in protists with small genomes, showing the complexity of infection by *Plasmodium* spp. in human patients and efficiency of this method in distinguishing highly virulent strains. Single‐cell WGS is yet to be applied to plant pathogens, but it has greatest perspective in understanding the function of unculturable unicellular pathogens such as members of the early diverging fungal lineages and alveolates (Ahrendt et al., 2018).

## 4 TARGETING ACTIVE ORGANISMS

DNA molecules are typically long‐lived and remain detectable for several weeks to months *post mortem*, depending on fragment length and habitat properties. Short (<200 bp) DNA fragments may be preserved for millennia in anoxic sediments and permafrost (Allentoft et al., 2012). Conversely, rRNA (including the ITS region) and mRNA of functional genes provide insights into the genes transcribed in a time frame of a few hours to a few days, effectively excluding organisms that are dormant (eggs, spores, sclerotia, cysts, etc.; Rajala, Peltoniemi, Hantula, Mäkipää, & Pennanen, 2011). However, sampling for RNA requires extra care and rapid pre‐treatment such as freezing in liquid nitrogen or storing in RNA preservation buffers, which is costly and sometimes inefficient (Rissanen, Kurhela, Aho, Oittinen, & Tiirola, 2010). Targeting RNA is, nonetheless, unavoidable in pathological studies of RNA viruses.

Alternative to RNA, microbial ecologists have used ^13^C (Radajewski, Ineson, Parekh, & Murrell, 2000) and the nucleotide analogue 3‐bromo‐deoxyuridine (Hanson, Allison, Bradford, Wallenstein, & Treseder, 2008) incorporation into substrate and assimilation into DNA of organisms that metabolize these enriched substrates. The ^13^C‐based stable isotope probing (SIP) is difficult to perform in natural conditions because of multiple carbon sources diluting the isotopic signal. This is especially relevant for eukaryotic pathogens that grow and accumulate ^13^C or nucleotide analogues into their DNA slowly and may use much of the labelled carbon for respiration. Nonetheless, ^13^C incorporated into DNA and fatty acids revealed flow of plant‐derived carbon through the soil food web and decline in pathogen‐to‐mycorrhiza ratio during secondary succession (Hannula et al., 2017). This method could be useful when addressing the pests and pathogens that use recent photosynthesis products amongst other organisms or detecting potential biocontrol agents.

## 5 TECHNOLOGICAL BIASES OF HTS METHODS

All molecular identification methods suffer from specific analytical biases. Marker bias may select for organisms that exhibit high copy numbers. Primer bias discriminates against targets that exhibit primer‐template mismatches, particularly in the 3´ end of the primer, which reduces their relative amplification efficiency by 1–2 orders of magnitude (Ihrmark et al., 2012; Tedersoo, Bahram, et al., 2015). Primer bias in the ITS region is important in several animal and plant pathogenic fungal groups (Tedersoo & Lindahl, 2016), nematodes and alveolates. PCR bias represents unequal amplification of target species due to differences in AT:CG ratio, DNA secondary structure and marker length (Ihrmark et al., 2012). Some arthropod and fungal groups exhibit introns in rRNA genes or long ITS1 or ITS2 regions, which may render corresponding taxa entirely unrepresented (Tedersoo, Bahram, et al., 2015). The best example concerns the ash dieback disease agent *Hymenoscyphus fraxineus *that exhibits a long 3′ terminal 18S intron, which renders the species undetectable using the ITS1F/ITSOF forward primers (see Cross et al., 2017). Most Oomycota possess a long ITS2 region, which may be discriminated against in studies targeting all eukaryotes (Riit et al., 2016). Method‐specific biases may occur in cloning and molecular identifier tag ligation procedures, where variants with highly skewed AT:CG ratios and specific terminal nucleotides may be favoured or discriminated against (Lindahl et al., 2013). Biases may differ among models and analysis chemistry in the same sequencing platform (Tedersoo et al., 2017).

Tag‐switches and chimeric molecules are common technical artefacts in HTS analyses. Chimeric molecules usually develop during PCR, when extension is incomplete (due to low processivity, short extension time or depletion of nucleotides) and these short fragments prime the templates during subsequent cycles. Chimeric molecules develop more commonly between two closely related organisms becoming more abundant with increasing number of PCR cycles and community complexity (Aas, Davey, & Kauserud, 2017; Haas et al., 2011). In essence, tag‐switch artefacts are also chimeric molecules that develop between multiplexed samples during the post‐PCR library preparation step (Schnell, Bohmann, & Gilbert, 2015).

## 6 PRACTICAL RECOMMENDATIONS FOR HTS‐BASED RESEARCH

### 6.1 Design of HTS studies

Study design depends on the objectives of research. Purely descriptive studies with haphazard sample collection and insufficient replication are difficult to publish and not worth the effort, except sequencing genomes or transcriptomes, or validating novel methods. Testing ecological hypotheses requires a proper well‐replicated sampling design. Many researchers seem to forget that technical replicates, multiple spatially autocorrelated subsamples and thousands of recovered OTUs do not serve as independent biological replicates (Prosser, 2010). This is particularly relevant in the geographically structured sampling with inherent hierarchical design and multilevel spatial autocorrelation.

One of the main questions in pathological and microbiological research is whether or not to pool subsamples. Pooling may strongly reduce analytical costs, but it also reduces small‐scale resolution. The answer depends on the research objectives, spatiotemporal scale and nature of the samples of the particular study. If individual samples (e.g., leaf, soil core) are small and expected to represent the community poorly, pooling multiple samples is a viable option. In case of hierarchical design (i.e., structured by block, plot or site), it is useful to pool multiple subsamples when the internal variation is not of interest. However, in most other cases, analysis of multiple independent samples is preferable due to the ability to estimate sampling error and address the importance of spatiotemporal variability. HTS analyses can easily recover slight shifts in taxonomic and gene composition; therefore, multivariate techniques require just 3–4 replicates to detect biologically important shifts in community composition (Balint et al., 2016). An extra replicate should be considered, because it is common to obtain low‐quality DNA or a limited number of sequences from some (typically 1%–10%) samples. Analysis of richness and diversity measures and pathogen load requires more samples, because univariate tests have lower statistical power.

### 6.2 Sample preparation for HTS analysis

HTS techniques are sensitive to spoiling, external contamination and cross‐contamination, hence requiring careful collection, handling and pre‐treatment to prevent contamination and overgrowth by fast‐growing moulds or DNA/RNA degradation (Lindahl et al., 2013). For pre‐treatment and storage, rapid air‐drying, freeze‐drying, deep‐freezing and fixing in preservation buffers work equally fine for DNA. Deep‐freezing and storage at −80°C works well for potential analysis of RNA, proteins and fatty acids for further analyses (Rissanen et al., 2010). Dried and frozen samples and frozen DNA solution can be usually preserved for decades with minor losses in quality.

To avoid contamination during various steps of analysis, it is recommended to divide the laboratory space into wet laboratory, DNA extraction and PCR laboratory, post‐PCR laboratory and HTS preparation laboratory. The PCR products from previous projects may represent the main source of contamination in HTS studies, because a single floating DNA molecule may be progressively amplified and sequenced. This can be controlled by efficient cleaning of laboratories using UV‐light and DNase‐containing solutions. Negative controls in all stages of analyses enable to detect and track contamination (Lindahl et al., 2013).

DNA or RNA extraction can be performed by using commercial kits or classical protocols developed since 1990s. Because extraction procedures may affect the recovered community composition (Lindahl et al., 2013), optimal protocols should be selected considering the mass, substrate and target organism (Brooks et al., 2015). Samples can be extracted at low overall mass (0.25 g), which recovers nearly comparable taxonomic richness with high‐quantity extraction (10 g) for microorganisms (Song et al., 2015). For small extraction quantities, it is relatively more important to thoroughly homogenize the sample. For ultra‐long amplicons and genomic analyses, bead beating should be kept at minimum duration. For metagenomic‐ and RNA‐based analyses, it is of particular importance to minimize the concentration of co‐extracted humic acids and saccharides that may interfere with downstream processes. The extracted DNA from soil and other organic‐rich substrates may require an extra purification step using filter columns or magnetic beads for optimal performance in (meta)genomics analyses (Bahram et al., 2018).

### 6.3 Marker and primer selection

For HTS‐based diversity analyses, it is very important to thoroughly consider the DNA/RNA marker based on desired taxonomic resolution. For routine community‐level analysis, species‐level resolution should always be targeted to avoid bulking together pathogenic organisms with closely related endophytes and saprotrophs (Critescu, 2014; Tedersoo & Nilsson, 2016). Nonetheless, strains of the same antagonist species may differ strongly in pathogenicity, which renders barcoding marker‐based detection of pathogens somewhat ambiguous. Bacterial species are typically identified based on the 16S rRNA gene, although this marker effectively enables operation at the subgenus and genus level (Yarza et al., 2014). The ITS region including the tRNA gene between 16S and 23S rRNA genes provides much greater taxonomic resolution, but its highly variable length and AT:GC ratio may generate biased views on composition particularly in HTS studies of prokaryotes (Benitez‐Paez & Sanz, 2017; García‐Martínez, Acinas, Antón, & Rodríguez‐Valera, 1999; Ruegger, Clark, Weger, Braun, & Borneman, 2014). Of eukaryotes, both fungi and oomycetes are routinely studied based on the ITS region, which has been established as a formal barcode for the latter group (Schoch et al., 2012). For protist kingdoms and Metazoa (animals), 18S and 28S rRNA genes, ITS and mitochondrial cytochrome C oxidase I subunit (COI) are all used, with preferences depending on particular groups considering taxonomic resolution and available reference material (Pawlowski, Audic, & Adl, 2012). The official animal barcode COI performs poorly in HTS analyses, because of the lack of conserved regions for inclusive primers and loss of primer‐template specificity with multiple degenerations (Geller, Meyer, Parker, & Hawk, 2013). The ITS region and 28S offer comparable species‐level resolution and allow use of strong primers in most animal groups such as Nematoda, Acari, Collembola and Hexapoda (Anslan & Tedersoo, 2015; Carneiro, Oliveira Lima, & Correia, 2017; Pacheco da Silva, Bertin, Blin, Germain, & Bernardi, 2014; Yang, Cai, & Cheng, 2011). Particularly for the ITS region, however, databases are sparsely populated with species‐ and genus‐level reference sequences from most arthropod, nematode and protist groups, because these have been excluded as classical barcodes at least partly due to difficulties with Sanger sequencing of heterozygous alleles and poor phylogenetic inference.

Guided by choice of a marker gene, selection of primers is important. Given the high sequencing depth, it is wise to focus on organisms in a broad perspective to secure inclusion of nearly all intended target taxa (Tedersoo & Lindahl, 2016). Because taxon‐specific primers are rarely all‐inclusive, it is therefore recommended to focus on all eukaryotes when addressing fungi, or Stramenopila when targeting Oomycota. For targeting pathogens in the tissue of plants or any other specific organisms, researchers may consider primers that exclude the host DNA or including additional blocking primers, especially if targeted pathogens are expected to occur at very low relative abundance. Supplied with 3′ terminal nucleotide modifications and in surplus concentration, blocking primers prevent annealing and elongation of host DNA marker by specifically binding to host DNA downstream of regular primers. This procedure is frequently used for mitochondrial DNA analyses (Vestheim & Jarman, 2008), but it is probably impossible to effectively design for nuclear rRNA genes. On the other hand, cotargeting host DNA enables to determine the relative abundance of pathogen DNA marker relative to host DNA marker that is comparable (i.e., without systematic bias) across samples.

Revisiting the coverage of widely used primers designed in early 1990s (White, Bruns, Lee, & Taylor, 1990) has revealed suboptimal performance and multiple unexpected mismatches to several taxa within the targeted group (Klindworth et al., 2013; Tedersoo & Lindahl, 2016; Tedersoo, Anslan, et al., 2015). While 1–2 non‐terminal mismatches are not important in sample preparation for Sanger sequencing, a single mismatch may result in underestimation of the taxon by 1–2 orders of magnitude (Ihrmark et al., 2012; Tedersoo, Bahram, et al., 2015). Accumulated sequence data have enabled molecular ecologists to construct more efficient degenerate primers or primer mixes for prokaryotes, fungi, oomycetes and animals (Apprill, McNally, Parsons, & Weber, 2015; Geller et al., 2013; Nilsson et al., 2018; Parada, Needham, & Fuhrman, 2016; Riit et al., 2016; Tedersoo & Lindahl, 2016; Tedersoo, Bahram, et al., 2016; Tedersoo, Liiv, et al., 2016; Toju, Tanabe, Yamamoto, & Sato, 2012). Table 3 provides a selection of high‐affinity primers recommended for use in HTS studies.

**Table 3:** Recommended primers for HTS‐based identification of pathogens and pests

Primer^1^	Marker: direction: position	Sequence	Target	Comment	Reference
*Prokaryotes*					
GM3F^a^	16S: fwd: 8	AGAGTTTGATCMTGGC	Bacteria	excl. minor phyla, Archaea	Muyzer, Teske, Wirsen, and Jannasch (1995)
515fB^b^	16S: fwd: 515	GTGYCAGCMGCCGCGGTAA	Prokaryotes	Microbiome projects	Parada et al. (2016)
806rB^b^	16S: rev: 806	GGACTACNVGGGTWTCTAAT	Prokaryotes	Microbiome projects	Apprill et al. (2015)
926r^b^	16S: rev: 926	CCGYCAATTYMTTTRAGTTT	Prokaryotes	Microbiome projects	Parada et al. (2016)
GM4R^a^	16S: rev: 1492	TACCTTGTTACGACTT	Bacteria	excl. minor phyla, Archaea	Muyzer et al. (1995)
SSU1492Fngs^c^	ITS: fwd: 1492	GTCGTMACAAGGTANCCG	Prokaryotes	excl. minor groups	This paper
189r^c^	ITS: rev: 189	TACTDAGATGTTTCASTTC	Bacteria	excl. minor phyla, Archaea	Hunt et al. (2006)
Eukaryotes (general)					
TAReuk454FWD1^d^	SSU: fwd: 565	CCAGCASCYGCGGTAATTCC	Eukaryote	BioMarks primer	Stoeck et al. (2010)
TAReukREV3^d^	SSU: rev: 970	ACTTTCGTTCTTGATYRA	Eukaryote	BioMarks primer	Stoeck et al. (2010)
1389F^e^	SSU (ITS): fwd: 1630	TTGTACACACCGCCC	Eukaryote, Prokaryote		Amaral‐Zettler, McCliment, Ducklow, and Huse (2009)
ITS9MUNngs^e^	SSU (ITS): fwd: 1635	TACACACCGCCCGTCG	Eukaryote	No prokaryotes	Tedersoo and Lindahl (2016)
1510R^de^	SSU: rev: 1780	CCTTCYGCAGGTTCACCTAC	Eukaryote	5′ poor, excl. Sordariomycetes, some nematodes	Amaral‐Zettler et al. (2009)
1510Rngs^de^	SSU: rev: 1780	WCBGCDGGTTCACCWAC	Eukaryote	No prokaryotes	This paper
ITS4ngsUni^efghi^	LSU (ITS): rev: 40	CCTSCSCTTANTDATATGC	Eukaryote	No prokaryotes	Tedersoo and Lindahl (2016)
TW13^efghi^	LSU: rev: 645	GGTCCGTGTTTCAAGACG	Eukaryote	Some prokaryotes; excl. dictyostelids	T.J. White, unpublished
TW14ngs^efghi^	LSU: rev: 960	CTATCCTGRGRGAAAYTTC	Eukaryote	excl. Microsporidea	Tedersoo and Lindahl (2016)
*Fungi*					
ITSOF‐T^f^	SSU (ITS): fwd: 90	ACTTGGTCATTTAGAGGAAGT	Fungi	excl. Mucoromycota, Saccharomycetes	Tedersoo et al. (2008)
ITS2ngs^f^	5.8S(ITS1): rev: 35	TTYRCKRCGTTCTTCATCG	Fungi	excl. minor groups	Tedersoo et al. (2017)
gITS7ngs^g^	5.8S (ITS2): fwd: 70	GTGARTCATCRARTYTTTG	Fungi	excl. minor groups	Tedersoo and Lindahl (2016)
LR5‐Fung^g^	LSU: rev: 880	CGATCGATTTGCACGTCAGA	Fungi, Metazoa, Stramenopila	Suited for living plant samples	Tedersoo et al. (2008)
*Oomycetes*					
ITS1Oo^h^	18S (ITS): fwd: 1795	GGAAGGATCATTACCACAC^2^	Oomycetes	incl. some Ochrophyta	Riit et al., (2016)
ITS3Oo^i^	5.8S (ITS2): fwd: 150	AGTATGYYTGTATCAGTGTC^2^	Oomycetes		Riit et al. (2016)
ITS5Oo^h^	18S (ITS): fwd: 1730	CTYRYCRTTTAGAGGAAGGTG	Stramenopila		This paper
ITS2Oo^h^	5.8S (ITS1): rev: 40	GCAGCGKTCTTCATCGRTGT	Oomycetes	incl. Synurophyceae	This paper
*Animals*					
jgLCO1490^j^	COI: fwd: 1490	TITCIACIAAYCAYAARGAYATTGG	Metazoa	excl. several groups, incl. Amoebozoa	Geller et al. (2013)
jgHCO2198^j^	COI: rev: 2198	TAIACYTCIGGRTGICCRAARAAYCA	Metazoa	excl. several groups, incl. Amoebozoa	Geller et al. (2013)

Notes

The newly reported primers have been designed to cover >99% targeted taxa and tested in silico and complex soil samples. Full set of primers used for bacteria, fungi, oomycetes and eukaryotes in general can be found in Klindworth et al. (2013), Nilsson et al. (2018), Riit et al. (2016) and Adl, Habura, and Eglit (2014), respectively.

^1^ Superscript letters indicate matching forward and reverse primer pairs.

^2^ Correct primer sequences compared to the trimmed ones in the original publication.

In analysis of complex pathological systems, food webs and diet of omnivores, organisms from multiple kingdoms can be targeted simultaneously. Strategies for this include a universal marker such as 18S rRNA or ITS for all eukaryotes, or different markers for each kingdom. Universal primers and primer mixes exist for the rRNA markers (Table 3). Different markers of similar length can be analysed in multiplex or mixed after separate amplification into a common library (de Barba, Boyer, Rioux, Coissac, & Taberlet, 2014). However, markers may yield >2 orders of magnitude difference in average sequencing depth (Tedersoo et al., 2017; Tedersoo, Anslan, et al., 2015), indicating that the relative discrimination factor should be considered beforehand. Metagenomic approach has proven a viable alternative for relative quantification of DNA of target organisms in soil (Bahram et al., 2018) and gut contents (Pearman et al., 2018).

One or both primers used for HTS should be tagged with a molecular identifier to enable multiplexing samples. These tags of typically 6–12 bases should differ from each other by at least 4 bases/indels (e.g., the “error‐correcting” Golay identifiers; Lundberg, Yourstone, Mieczkowski, Jones, & Dangl, 2013) to prevent random mutations in tags or impure synthesis to erroneously switch sequences among samples. The tagged primers may also include a platform‐specific sequencing primer, but such long oligonucleotides may perform poorly (Lindahl et al., 2013). For Illumina sequencing, 96 combinations of Nextera indexes can be ligated by PCR. Primers tagged with identifiers only are cheaper and can be used for analysis employing any sequencing platform, rendering these usable for many years. To reduce the competition among tagged amplicons in the ligation step, it is advisable to select all identifiers to start with the same nucleotide and use a 2‐base linker sequence with no match to any of the templates. Identifier tags that have an AT:CG ratio less or more than 0.25–4 tend to perform poorly (Tedersoo & Nilsson, 2016). It is strongly recommended to add identifier tags to both the reverse and forward primers to minimize the tag‐switching (Gohl et al., 2016).

### 6.4 PCR

Prior to PCR, it is recommended to quantify DNA and use equal amounts of template for each sample to be able to use the same number of PCR cycles across the study (Gohl et al., 2016). The PCR mix should include a high‐affinity and high‐processivity polymerase (e.g., Pfu, Phusion, Q5) to minimize incorporation of erroneous nucleotides and generation of partial reads that can be converted to chimeric sequences in subsequent extension cycles. These more expensive polymerases strongly reduce the number of chimeric sequences and artificial taxa comprised of error‐infested sequences (D'Amore et al., 2016; Gohl et al., 2016). For HTS analysis, the primer annealing temperature could be reduced by ca 5°C compared to regular PCR to promote amplification of templates with one or two mismatches to primers. The number of PCR cycles should be kept at minimum—so that a relatively weak band or smear of suitable size is seen on an agarose gel. Increasing extension time is also likely to reduce incomplete amplification and hence chimera formation (D'Amore et al., 2016; Lindahl et al., 2013). Low input DNA content results in lower amount of inhibitors and less chimeric sequences (D'Amore et al., 2016; Gohl et al., 2016). Due to stochastic variation, it is recommended to use at least two PCR replicates that can be pooled post‐amplification (Alberdi et al., 2018; Lindahl et al., 2013; Tedersoo et al., 2010).

Amplicon purification depends on further analyses and choice of sequencing platform. It is recommended to normalize amplicon concentration across samples to reduce variation in sequencing depth among samples several‐fold (Lindahl et al., 2013). The equimolarly mixed amplicons are subjected to platform‐specific adapter ligation in the library preparation step. It is recommended to order library preparation from a sequencing service provider to secure their quality standards and leave the risk of failure to the service provider. Researchers should check the quantity and quality requirements from each service provider, because these may differ greatly. The quantity appears to be negotiable, because service providers usually request 5–10 times more material than they use. Due to high demand, it takes typically 1–2 months to receive the sequences. It does not pay off to order bioinformatics service, because companies provide standard quality,.fasta‐ and.fastq‐formatted files. These can be handled using custom options in any bioinformatics platform, whereas the service provider's analysis routine may be suboptimal (i.e., optimized for bacterial 16S rRNA gene, mouse or human samples) or untransparent.

### 6.5 Controls and technical replication

To quantify contamination and technical artefacts such as sequencing errors, chimera formation and tag‐switching, it is recommended to run three types of control samples in parallel. Negative control samples should be incorporated during various steps of sample processing (DNA/RNA extraction, PCR; Lindahl et al., 2013). One or more positive controls—preferably organisms not expected in studied communities—might be included to quantify tag‐switching (Schnell et al., 2015; Tedersoo et al., 2017). Mock community of known composition may provide information about the rates of chimera formation and efficiency of recovering ingredient taxa (Nguyen, Smith, Peay, & Kennedy, 2015). It is possible to use artificial DNA molecules for positive control and mock community samples, because their length, AT:CG ratio and homopolymer content can be controlled and their concentration can be precisely determined (Palmer, Jusino, Banik, & Lindner, 2017).

Technical replication is unnecessary in most cases, because these observations cannot be used as independent data points in the analysis. However, limited technical replication of a few samples may be feasible to estimate the performance and reproducibility of the method especially for newly developed protocols (Alberdi et al., 2018; Brooks et al., 2015).

### 6.6 Quality filtering of HTS data

Analysis and quality filtering of HTS data are by far more challenging than viewing and editing Sanger sequencing reads because of large amounts of data and no clearly readable chromatograms. There is a myriad of available software for bioinformatics data analysis, most of which, such as mothur (www.mothur.org) and QIIME (www.qiime.org), are run on command line. Both of these popular bioinformatics platforms are optimized for alignment‐based bacterial 16S rRNA gene analysis. QIITA (https://qiita.microbio.me) is a recently developed web‐based analysis platform for bioinformatics and analysis workflow optimized for bacteria‐targeted and metagenomic research. PipeCraft is a user‐friendly software with a graphical interface, multiple options incorporated from other programs, capacity to analyse metabarcoding data from all sequencing platforms and compatibility with Linux, Mac and Windows, which all attract non‐bioinformatician users (Anslan, Bahram, Hiiesalu, & Tedersoo, 2017). For analysis of non‐alignable markers such as ITS, PipeCraft outperforms other bioinformatics pipelines in terms of input data formats, available analysis options and output quality (Anslan et al., 2018). Comprehensive overview about available analysis platforms for amplicon and metabarcoding data is given in Oulas et al. (2015). Bioinformatics analysis of fungal data is reviewed in Nilsson et al. (2018).

Although the output of HTS platforms is converted to the same format, these data differ in the distribution of errors and require different options for analysis (Anslan et al., 2017; Knief, 2014; Laehnemann, Borkhardt, & McHardy, 2015; Reuter et al., 2015). Quality‐trimming is the first step of bioinformatics analysis and it is usually based on removing the 3′ end of sequences (or entire sequences) if it falls below specific quality threshold, the optima of which differ by sequencing platform. In a simultaneous sample demultiplexing process, sequences are re‐assigned to biological samples based on the molecular identifiers. For demultiplexing sequence data with Golay barcodes, we recommend allowing 1–2 mismatches to tags and 1–2 mismatches to primers to account for random errors and natural primer‐template mismatches. Demultiplexing from a single end typically enables to recover 40%–70% of all reads, but approximately a quarter of these are lost when accounting for the other tagged primer as well (Tedersoo et al., 2017). However, dual‐tag demultiplexing enables to remove tag‐switching artefacts and incomplete sequences (Kozich, Westcott, Baxter, Highlander, & Schloss, 2013).

To reduce computation time, quality‐filtered sequences are usually pre‐clustered using 100% or 99% identity and subjected to chimera checking. Chimera detection performs best when combining de novo and reference‐based methods as implemented in UCHIME (Aas et al., 2017; Edgar, Haas, Clemente, Quince, & Knight, 2011) that can be run in all bioinformatics platforms.

Extraction of variable region(s) may precede or follow chimera check. Extraction of ITS and other variable regions in rRNA genes enables simultaneous removal of non‐target organisms and focus on a shorter but more variable barcode that has improved taxonomic resolution (Bengtsson‐Palme et al., 2013; Hartmann, Howes, Abarenkov, Mohn, & Nilsson, 2010).

### 6.7 Sequence clustering and Operational Taxonomic Units

Quality‐filtered and trimmed sequences are subjected to clustering into OTUs, for which multiple algorithms exist (Kopylova et al., 2016). The best results are obtained when using open‐source de novo clustering with single‐linkage algorithms (Frøslev et al., 2017; Lindahl et al., 2013). Except for Illumina data, it is recommended to collapse homopolymers to trimers for clustering (Lindahl et al., 2013) or lowering the gap extension penalty, because other platforms are sensitive to indels in homopolymers. Although many protocols recommend removing sequences containing homopolymers of >8 or >10 bases, we do not encourage this practice for the non‐coding regions, because many organisms do have naturally long homopolymers in these markers (Potter et al., 2017; Tedersoo et al., 2017).

In spite of different taxonomic resolution, the bacterial 16S and eukaryote 18S, 28S, ITS and COI sequences are typically clustered at 97% sequence identity, which is regarded as a compromise between natural intraspecific and interspecific sequence variation and random sequencing errors. The 97% sequence similarity threshold for all of these marker genes (except COI in some groups) is too conservative for species‐level identification of most taxa. For example, some biological species of *Fusarium* display no variation at all in the relatively unconserved ITS region (Park et al., 2011). Therefore, HTS analysis of the ITS + 28S rRNA gene (Walder et al., 2017) and transcription elongation factor 1 subunit α (TEF; Karlsson et al., 2016) have been used to specifically distinguish *Fusarium* spp. With low‐resolution markers, analysis of exact sequence variants can be performed using 100% similarity threshold or the DADA2 clustering program (Callahan et al., 2016).

All clustering methods generate more OTUs than expected at any barcoding threshold with increasing sequencing depth, indicating accumulation of PCR and sequencing errors into rare “satellite” taxa (Frøslev et al., 2017). This can be ameliorated by performing two or more consecutive clustering steps (Nguyen et al., 2015), post‐clustering removal of taxa based on co‐occurrence or phylogenetic algorithms (Frøslev et al., 2017; Potter et al., 2017) or focus on longer DNA fragments, where random errors are evened out (Tedersoo et al., 2017). It is further recommended to remove global singletons and perhaps OTUs with <5 or <10 sequences, depending on sequencing depth, as potentially artefactual (Frøslev et al., 2017; Lindahl et al., 2013; Nguyen et al., 2015; Tedersoo et al., 2010).

### 6.8 Sequence‐based taxonomic identification and taxon communication

Selection of one or more reference databases is essential for sequence‐based identification (reviewed in Kashyap, Rai, et al., 2017b). Since up to 20% of the material in INSDc is of poor quality or misidentified, initiatives such as UNITE (https://unite.ut.ee/), SILVA (www.arb-silva.de) and UniEuk (https://unieuk.org/) have generated databases and reference data sets populated with filtered and third‐party annotated sequences. SILVA is focused on nuclear SSU and LSU sequences of prokaryotes and eukaryotes, but both oomycetes and fungi are poorly represented and have problems with taxonomic assignment (Tedersoo Tooming‐Klunderud et al., 2017; Tedersoo, Anslan, et al., 2015; Yarza, Yilmaz, Panzer, Glöckner, & Reich, 2017). The UniEuk initiative is focused both on taxonomy and on curation of high‐quality 18S rDNA sequences of eukaryotes (Berney et al., 2017). The current version of UNITE includes SSU, ITS and LSU sequence data for all eukaryotes, although only fungal and oomycete ITS sequences have been intensively annotated for taxonomy, sequence quality and ecological metadata. Some pathogenic fungal groups, in particular, have been thoroughly checked, annotated and assigned for type status in unite (Nilsson et al., 2014). In the bold database (Ratnasingham & Hebert, 2007), curated COI sequence data for animals, Oomycota and other specific groups of protists are maintained. Thus, these databases provide best‐suited species‐level reference data for general molecular identification of pathogenic organisms. However, researchers focused on more narrow groups such as *Fusarium* or *Phytophthora* could use Fusarium‐ID (Park et al., 2011) and the Phytophthora Database (www.phytophthoradb.org) in addition. Animal and human pathogens have annotated sequence data in the ISHAM‐ITS database (Irinyi et al., 2015). Metagenomic and metatranscriptomic analyses require inclusion of functional gene and genomics databases for combined taxonomic and functional analysis (Huson, Mitra, Ruscheweyh, Weber, & Schuster, 2011). Detection of viruses amongst genomic, metagenomic and metatranscriptomic reads requires some specific data mining effort. Pipelines such as VirusFinder (Wang, Jia, & Zhao, 2013) and particularly VirFind (Ho & Tzanetakis, 2014) enable to search for virus motifs from custom sequence data sets and EST databases. As a reference for identification, virologists use mostly the RefSeq database of INSDc (O'Leary et al., 2016) and Comprehensive Phytopathogen Genomics Resource (CPGR) database (Hamilton et al., 2011).

For taxonomic assignments, it is most common to use BLAST‐based similarity search methods for representative sequences of each OTU (Nilsson et al., 2014; Tedersoo & Nilsson, 2016). The Naive Bayesian Classifier (Porras‐Alfaro, Liu, Kuske, & Xie, 2014; Wang, Garrity, Tiedje, & Cole, 2007) is widely used for conservative identification in prokaryotes, but this method has gained little popularity among mycologists due to a low proportion of taxa identified to species or genus level. This has been improved in ProTax‐Fungi, which provides statistical assessment of assignment precision to different taxa from species to phylum ranks (Abarenkov et al., 2018). In fungal and oomycete ITS sequences, species, genus, family and order levels can be approximately approximated at >97%–99%, >90%, >85% and >80% ITS sequence similarity, respectively, to the closest identified sequence (Tedersoo et al., 2014; Tedersoo et al., 2017; data in Riit et al., 2016). In bacterial 16S (full‐length), these figures are >98%–99%, >94.5%, >86.5% and >82%, respectively (Yarza et al., 2014). Due to different rates of rRNA gene evolution, there are multiple exceptions, with sordariomycete (Ascomycota) and oomycete species tending to exhibit greater similarity and early diverging fungal groups lower similarity. These differences are even greater among animal and protist groups (Anslan & Tedersoo, 2015; Nassonova, Smirnov, Fahrni, & Pawlowski, 2010; Yang et al., 2011), but remain still poorly understood for most taxa (Pawlowski et al., 2012).

An optional step is to assign functional traits such as pathogenicity information to OTUs, for which database‐related tools exist. For Bacteria, an automated pipeline SINAPS (Edgar, 2017) enables to search and predict custom traits using the ProTraits reference database (Brbic et al., 2016). The basic fungal traits can be assigned to taxonomic profiles using a tool in FunGuild database (Nguyen et al., 2016). Its main limitation is genus‐level operation, although it alerts that many genera contain both pathogens and saprotrophs or endophytes. As discussed above, the detected “pathogenic” OTUs may be non‐pathogenic on non‐hosts, rendering the assignments strongly context dependent. Therefore, more accurate metadata with host‐ and tissue‐related traits assigned to species, species hypotheses (see next paragraph) or isolates/sequences are urgently needed.

HTS studies enable to recover tens of thousands of OTUs, most of which cannot be usually assigned to described species, which renders these difficult to communicate across studies. The unite and bold databases use taxon codes (species hypotheses and BINs, respectively) linked to Digital Object Identifiers (DOIs). These machine‐readable DOIs enable communication of both named and unnamed taxa across studies and time (Kõljalg et al., 2013; Kõljalg, Tedersoo, Nilsson, & Abarenkov, 2016; Ratnasingham & Hebert, 2013).

### 6.9 Post‐bioinformatics data quality control

For soil and raw tissue samples with non‐optimal storage conditions, it may be important to estimate sample quality due to potential overgrowth by moulds (Lindahl, Boer, & Finlay, 2010). This can be performed by measuring the average size of extracted DNA/RNA molecules on the gel or calculation of the relative abundance of moulds (fungal orders Hypocreales, Mucorales, Umbelopsidales and Mortierellales). Dominance of a single mould OTU, which is usually associated with reduced taxonomic richness, can be considered indicative of sample spoilage (Tedersoo et al., 2014).

Similarly, it may be feasible to exclude samples with <5‐ to 10‐fold less sequences compared with the median. Such poor recovery may be ascribed to the failure to normalize a sample, poor performance of particular identifier tags and/or dominance of particular organisms in a sample, which are disfavoured in the library preparation, sequencing or quality‐filtering steps. In spite of attempts to normalize quantity of amplicons, the number of retrieved sequences typically vary >3‐fold. It is common to rarefy all samples to the same minimum sequencing depth, but this loses vast majority of taxonomic information. Therefore, it is recommended to calculate residuals of richness relative to square‐root or logarithmic function of sequencing depth (whichever fits better), or use these functions as covariates in uni‐ and multivariate statistics (Balint et al., 2016).

Due to high sensitivity, HTS data commonly suffer from traces of environmental contamination or tag‐switching (see above). Information about the OTUs in control and experimental samples enables evaluation of these technical biases and need for extra quality filtering (Nguyen et al., 2015; Palmer et al., 2017). In case of extensive tag‐switching, sequences can be removed according to statistical formulae (Larsson, Stanley, Sinha, Weissman, & Sandberg, 2018). Although the tag‐switching artefacts usually account for 0.1%–3% of all sequences (Palmer et al., 2017; Schnell et al., 2015; Tedersoo et al., 2017), these may blur qualitative diversity analyses and particularly network analyses that are sensitive to adding low‐abundance OTUs. More importantly, tag‐switching may generate false‐positive implications of low‐level presence of a pathogen or biocontrol organism, especially when these dominate some samples in the library.

### 6.10 HTS data analysis

HTS platforms generate enormous OTU‐by‐sample data matrices, which cannot be sometimes fully loaded into common spreadsheet programs. Therefore, experts use python or perl scripts to navigate and transform the data in text format. These large community matrices also test the limits of statistical software and processors. Many commonly used methods for community phylogenetics, bootstrap resampling and network analysis become computationally prohibitive. Thus, use of computation‐efficient algorithms is warranted. To reduce the computation requirements, the data can be compressed by removal of rare species, which typically reduces unexplained variance and promotes statistical power (Põlme et al., 2018), but its effect on potential type I and type II error is not known in multivariate or network analyses.

For multivariate analyses, we recommend downweighing abundant OTUs by Hellinger (square‐root) transformation to account for the semiquantitative nature of HTS. Use of qualitative binary data (presence/absence) is not recommended, because of lower fit due to loss of the (semi)quantitative information and artificial equalization of potentially artefactual low‐abundance (including tag‐switch artefacts) and real high‐abundance OTUs (Balint et al., 2016). We recommend use of PERMANOVA for explicit statistical testing of shifts in community composition, because it allows including interactions, random factors and nested designs. ANCOM and Random Forest machine‐learning algorithm provide statistical information about the performance of each OTU in the community matrix. General information about multivariate analysis methods suitable for HTS data is given in Buttigieg and Ramette (2014). Notably, the same multivariate techniques are commonly used to analyse standardized microarray and metagenomic and metatranscriptomic data (Thomas, Gilbert, & Meyer, 2012).

In univariate analyses, OTU richness, diversity, colonization, damage and relative abundance of certain taxonomic or functional groups are used as dependent variables. Apart from considering sequencing depth and treatment of rare OTUs, the analyses should follow best statistical practices including appropriate transformations, testing assumptions, etc. Balint et al. (2016) provide an overview about general recommendations to statistical analysis of HTS data, computation‐efficient programs and potential pitfalls.

### 6.11 HTS data storage and reporting

HTS data sets are stored both as raw data files and elaborated data sets. The raw.fastq files, metadata files and files with identifier tag and primer information are kept in the Short Read Archive (SRA). These files enable users to perform all steps of bioinformatics analyses including generation of OTU table and identification. This is important from several aspects such as confirming earlier findings with updated filtering procedures, addressing additional questions and performing metastudies using standardized filtering procedures. It is, however, discouraged to submit representative sequences of HTS‐derived OTUs to public databases because of their short length, potentially artefactual nature and unreliable taxonomic annotation. These environmental sequences would increase the proportion of poorly annotated and erroneous data and complicate identification in subsequent studies.

Curated OTU‐by‐sample matrices including technical and environmental metadata, representative sequences as well as taxonomic and functional annotations should be deposited in machine‐readable FAIR data format in specific data repositories such as Dryad Digital Repository (www.datadryad.org) and DataOne (www.dataone.org). Darwin Core (https://rs.tdwg.org/dwc/) is the main standard for biodiversity data, which is linked to MIxS (https://gensc.org/mixs/) and MIMARKS (https://wiki.gensc.org/index.php?title=MIMARKS) standards by the Genomics Standards Consortium that are relevant to all gene sequence data and marker gene sequence data, respectively. Metagenomic data should follow MIxS and MIMS (https://wiki.gensc.org/index.php?title=MIGS/MIMS) standards (ten Hoopen et al., 2017). The machine‐readable FAIR data format allows researchers to understand and rapidly incorporate the data into meta‐analyses. Such standardized data sets in digital repositories enable separate DOI‐based citations.

In publications, it is important to refer to any additional data in the supplement or data repositories. It is also important to record and describe precisely all analytical steps including specific options in data filtering, because this information provides important details about the data quality and stringency of filtering to the readers. Nilsson et al. (2011) provide thorough recommendations about the required details for molecular and bioinformatics analyses.

## 7 PERSPECTIVES

Only a fraction of available high‐throughput identification potential has been currently used in plant pathology. This is related to the practical surveillance‐oriented work of plant pathologists and entomologists but focus on human and animal subjects by molecular pathologists. Governmental plant health surveillance organizations need to follow certified protocols for diagnosis, which develop slowly due to time‐consuming tests. Limited budgets also hinder the possibility of purchasing high‐throughput analysis equipment by governmental institutions. Considering analysis costs and time, practicing pathologists would certainly take advantage of qPCR/ddPCR for real‐time quantification of specific pathogens and custom microarrays for simultaneous detection and quantification of multiple selected pathogens. In the nearest future, it may be possible to detect multiple organisms including pathogens using high‐throughput sequencing on portable pocket‐size sequencers as demonstrated for viruses using the Oxford Nanopore MinION platform (Loman et al., 2015). For a simplified procedure, a single working day is essentially required for sample collection, analysis and interpretation of results. Detection of organisms may occur much faster using nanotechnological biosensors that recognize multiple specific volatile molecules in parallel using antibody receptor‐based optical or electrochemical detection (Kashyap, Kumar, & Srivastava, 2017a; Khater, Escosura‐Muñiz, & Merkoçi, 2017; Sutarlie, Ow, & Su, 2017). Throughput of these biosensors can be greatly increased by using a microchip format for signal detection (Wang, Long, Liu, Wu, & Hu, 2017).

Other high‐throughput identification methods are more time‐consuming but also more sensitive and thus better suited for research purposes. Metagenomic and metatranscriptomic methods offer great potential when targeting viruses (Zhang et al., 2005) or these together with prokaryote and eukaryote pathogens and pests simultaneously (Chandler, Liu, & Bennett, 2015). Alternatively, nematodes, insect pests, oomycetes and fungi can all be assessed by using a mixture of degenerate primers targeting the same marker or multiplex primers targeting different markers via metabarcoding (de Barba et al., 2014; Tedersoo, Bahram, et al., 2016; Tedersoo, Liiv, et al., 2016). Targeted template capture by use of specific hybridization probes and immunochemical methods allows concentrate marker genes and pathogenesis‐related genes of antagonists (Dowle, Pochon, Banks, Shearer, & Wood, 2016) that can be further identified using PCR‐free methods.

Because of great intraspecific resolution, high‐throughput fingerprinting and population genomics approaches offer enormous potential for diagnosis of aggressive strains or pathotypes and uncover their patterns of dispersal (O'Hanlon et al., 2018) and potential hybridization (Qiu, Cai, Luo, Bhattacharya, & Zhang, 2016). Given appropriate quality filtering, these methods are sensitive enough to distinguish rare alleles and SNPs from noise (Isola et al., 2005) in hundreds of samples in parallel. Whole‐genome sequencing and transcriptome analyses complement HTS‐based identification methods by shedding light into pathogenesis mechanisms and facilitating generation of vaccines and biocides and selection of biocontrol agents (Grünwald et al., 2016).

For correct identification, community‐curated and taxonomically annotated reference databases are urgently needed. Such databases are maintained only for a few most important pathogen groups and cover the main barcoding marker genes (Park et al., 2011, 2008). Sequence databases should share third‐party metadata and taxonomic annotations, whenever these are updated in one of these (Nilsson et al., 2014). In spite of a large proportion of erroneous data, INSDc will certainly continue to play a central role in bridging more specific databases encompassing genes from all domains of life. So, let's contribute well‐annotated and high‐quality sequence data to INSDc to benefit the pathologists research community! This also applies to HTS data sets and data matrices, the great practical and scientific value of which can be recognized perhaps after several decades. Alongside storing sequence data, it is important to maintain tissue and soil samples that can be resource efficiently kept dried at room temperature. In spite of small size, accumulating DNA samples tend to rapidly fill refrigerators in entire rooms and are vulnerable to technical failure and power cuts. Besides the possibility of morphology‐based re‐identification and description of new species, both botanical and pathological herbaria provide excellent sources to trace back the evolution and dispersal of pathogens and pests (Drenkhan et al., 2017; Drenkhan, Riit, Adamson, & Hanso, 2016; Yoshida et al., 2014).

Taken together, high‐throughput identification techniques offer great promise for detection and rapid identification of new pathogens and diseases in humans as well as tree and crop plantations and early warning systems such as the sentinel nurseries and botanical gardens. HTS has already demonstrated its usefulness in studies of soil‐ and plant‐associated microbial communities for detection of new potential pathogens and potentially invasive species before their introduction to the new environment and contact with new hosts. We predict that rapid monitoring methods such as nanopore sequencing, microarrays and nanotechnological biosensors will become particularly useful for early disease diagnostics and smart application of countermeasures such as biocides and biocontrol agents.

## AUTHOR CONTRIBUTIONS

M.C., L.T. and R.D. developed the concept; S.A. constructed the figure and contributed specific details together with C.M.‐R.; and L.T. wrote the article with input from co‐authors.

## Data Availability

This review does not contain original data; all compiled published data are available in the main text body and tables.
